# Social Position of the Veterinarian1Read at the semi-annual meeting of the Schuylkill Valley Veterinary Medical Association, Reading, September 7, 1896.

**Published:** 1896-10

**Authors:** J. W. Sallade

**Affiliations:** Pottsville, Pa.


### THE JOURNAL OF COMPARATIVE MEDICINE AND VETERINARY ARCHIVES.

VOL. XVII. *OCTOBER*, 1896. No. 10.

### SOCIAL POSITION OF THE VETERINARIAN.[1]

BY J. W. SALLADE, V.S.,
POTTSVILLE, PA.

THE veterinarian, being a professional man, rightfully belongs to the more cultivated portion of a community. He should, as a learned man, command a high social as well as a useful position; and it may not be egotism to claim for him a distinctively lofty position in all that relates to the social improvement of the community. In social science, education, health, and the arts, it is his function to participate in all discussions calculated to advance the interests of society. In order to creditably occupy this lofty position in society it is essential that the veterinarian be a man of high character, education, and energy, and possess a ceaseless determination to elevate himself, and thereby his profession, in the estimation of the community, and thus place himself on the top round in the social swim. He should keep well posted at all times in matters of national, State, and local politics; so also as regards other subjects likely to affect the welfare, interests, and credit of the community. He ought to be a walking encyclopædia, ever ready to express himself intelligently, no matter what the topic of conversation may be.

The young veterinarian, if possessed of a proper preliminary education and a thorough college training, will not find it difficult, at this late day, to enter upon the full discharge of his social as well as his professional obligations. Times in America

[1] Read at the semi-annual meeting of the Schuylkill Valley Veterinary Medical Association, Reading, September 7, 1896.

have changed. Instead of the veterinarian being classed with the "horse doctor" and "cow leech" of bygone days (for those once useful members of society have yielded their existence to the inevitable fate that was born to them and have ceased to be factors in our noble profession), the veterinarian of to-day ranks among the learned professions, and is recognized by all as a benefactor to human society. Not only do the domestic animals switch their tails with satisfaction and as a mark of appreciation upon the veterinarian, when in distress, but the community recognizes also that its own health depends upon the health of its dumb friends. This altered condition of affairs, therefore, makes it comparatively easy for the veterinarian of to-day to gain social prominence. He cannot fail to attract attention lest there should be danger that his constant association with a less intelligent class of animals should exercise a degrading influence upon his mental faculties, but unless he was born a calf he will never wear horns.

It should be the veterinarian's constant aim to serve the community intelligently both as veterinarian and civilian. He must take a deep interest in the health of the people, look up carefully all sanitary irregularities, and devote his best offices to the correction of the same; he must be an educator, and teach hygiene and sanitary laws to the people, and this he must be willing to do for the good of the community, depending upon social and professional recognition for his fee. The veterinarian should be amiable, affable, firm, and decisive without being arrogant in his assumptions, and thus impress his importance and individuality upon the daily record of the community. While his attainments should fit him to associate with the cultivated and literary portion of the community, he must not forget that his duty to the people entails obligations requiring that he adapt himself to all classes under varying circumstances; and therein the social position of the veterinarian assumes a distinct and peculiar form, likened unto that of the politician. In his daily intercourse with the people he meets as many dispositions of mind as microbes in a tubercular section. He must associate with all and apply his own resources to suit the case in hand; tendency is a strong determination toward some particular mode of action. A man's inclinations are variable, but if the veterinarian cultivates a tendency to accommodate he will ultimately prevail. In direct proportion to his usefulness and value in a community will he be able to command the respect and admiration of the people. The veterinarian should be self-possessed and dignified at all times, prudent in the use of language, civil to all, conservative in self-laudation.

Considering the veterinarian individually, ascribing to him proper qualities of mind, with an honest intention to discharge his duties to himself, his profession, and the community, recognizing the importance of his vocation and his loyalty to principle, who will deny him social distinction? Collectively taken, the veterinary profession comprises some of the most learned, brightest, and foremost scientists. Have you ever heard of a man or a class of men who have made great discoveries or distinguished themselves in the field of science or art who did not have the social gates opened and been invited to green pastures?

We must never lose sight of the fact, however, that much depends upon individual effort. No one should be so base as to expect recognition simply because he represents a noble profession, and if he should, he will find himself an object of pity, ostracized and banished in disgrace. The object and aim of every true citizen are to advance, and it should also be the aim of the veterinarian to constantly raise himself. In order to do this, if he would attain a high rank socially in the community or professionally among his colleagues, he should ever bear in mind that he must furnish some equivalent. High social position may only be attained by those born with a silver spoon in their mouths and those possessing purity of character and ability in the sphere of life representing a useful career. The one is obtained without merit, while the other represents natural attainment; natural because nine times out of ten it is acquired by self-endeavor.

Few enter upon the study and practice of veterinary medicine whose social position has been established by birthright. Almost every devotee is a volunteer from the rank and file of the ordinary citizen; imbued with the spirit of love for right he enters upon a sphere of life now acknowledged under all the existing circumstances to be, from a financial point of view, an uninviting field of labor. Yet conscious of the fact that a community appreciates an offering so vital to his physical existence, the veterinarian approaches the goal and offers his best and most patriotic services to the community, endeavoring by his unselfish efforts to merit professional recognition and social standing.

There is no other profession that can justly claim or maintain, by just and right means, a more direct and influential position for good in a community than that of the veterinarian. The dentist may supply a lost molar, the lawyer may draw a mortgage or plead a case in court, the preacher of the gospel may exhort and tell of all that is good and bad and point to the imaginary road to the kingdom, but it remains for the veterinarian to give an unadulterated food-supply, an honest beefsteak, and a pure glass of milk, articles entering so largely into the necessaries of life, to maintain and sustain growth and development. Possessing all these high prerogatives, we claim that the social position of the veterinarian is among the highest known to mankind, distinctively elevating, and one that may only be occupied by men who, first of all, possess the requisite inherent qualifications and faculties, and then having those latent powers cultivated and groomed until they expand in magnificent proportions, blooming forth like June roses, with all their fragrance permeating the very anatomy of humankind. Having attained this pinnacle of greatness, gentlemen, then and only then may you rest conscious that you are occupying the true social position allotted by God and the sciences and the arts to the true veterinarian.

